# Risk Factors and Prevalence of *Helicobacter pylori* Infection in Persistent High Incidence Area of Gastric Carcinoma in Yangzhong City

**DOI:** 10.1155/2014/481365

**Published:** 2014-01-14

**Authors:** Yangchun Zhu, Xiaoying Zhou, Junbei Wu, Jing Su, Guoxin Zhang

**Affiliations:** ^1^Department of Gastroenterology, People's Hospital of Yangzhong County, Yangzhong 212200, China; ^2^Department of Gastroenterology, The First Affiliated Hospital of Nanjing Medical University, Nanjing 210029, China; ^3^Department of Gastroenterology, Shengze Hospital of Nanjing Medical University, Wujiang, Jiangsu, China

## Abstract

*Aim*. The aim of this study was to investigate the prevalence and risk factors of *H. pylori* infection in areas with high prevalence of gastric cancer in Jiangsu Province, China. *Methods*. A prospective epidemiologic survey of *H. pylori* infection was accomplished in a natural population of 5417 individuals in Yangzhong city. Questionnaires and 13C-urea breath test for *H. pylori* infection were performed. *Results*. Among 5417 subjects who completed questionnaires and 13C-urea breath test, 3435 (63.41%) were *H. pylori* positive. The prevalence reached a peak at the age of 30–39 years (90.82%). There was significant difference between sexes and women had a higher infection rate than men. The prevalence of *H. pylori* infection was also associated with eating kipper food and fried food. No association between *H. pylori* prevalence and smoking or drinking was found. Compared to healthy individuals, people with dyspeptic diseases (peptic ulcer, gastroenteritis) presented a high prevalence of *H. pylori* infection. Using multivariate logistic regression analysis, age and history of peptic ulcer and gastroenteritis were the independent predictors for *H. pylori* infection. *Conclusions*. Yangzhong city had a high prevalence of *H. pylori* infection and was related to several risk factors. The underlying mechanisms are needed to be further investigated.

## 1. Introduction

*Helicobacter pylori* is a microaerophilic Gram-negative spiral bacterium [1]. Its helix shape is thought to have evolved to penetrate the mucoid lining of the stomach [2]. It is linked to the development of chronic gastritis, gastric ulcers, duodenal ulcers, and stomach mucosal atrophy. Moreover, *Helicobacter pylori* is well recognized as a class I carcinogen because chronic inflammation and atrophy can further lead to malignant transformation [3, 4]. At least half the world's population is infected by this bacterium, making it the most widespread infection in the world, especially in the developing world where rates are estimated to be around 80% [5].

*H. pylori* is contagious, although the exact route of transmission is not known [6, 7]. Person-to-person transmission by either the oral-oral or fecal-oral route is most likely. *H. pylori* may also be transmitted orally by means of fecal matter through the ingestion of waste-tainted water [2]. Many of the reported factors for *H. pylori* infection included poor hygiene, deficient sanitation, and crowded living conditions [8]. However, the roles of many other factors associated have not been elucidated.

The aim of the current study was to determine the prevalence of *Helicobacter pylori* in the census population in Yangzhong city of Jiangsu Province, where there is a high prevalence of gastric cancer, and to assess the risk factors for *Helicobacter pylori* by an extended anamnesis, involving data on sex, age, educational level, smoking, drinking, as well as dietary factors.

## 2. Methods

### 2.1. Study Population

A total of 5417 healthy individuals aged between 30 and 69 years old from six rural villages in Yangzhong counties, northern Jiangsu province, from August 2009 to October 2011 underwent a comprehensive medical survey at the Center for Preventive Medicine in Yangzhong hospital as part of a survey study. There are seven administrative sub-autonomous regions in Yangzhong city, 77 counties in total. We assigned a consecutive number from 1 to *N* numbering counties of each region. Then we used a computer programme randomly chose one county from each region, except which population is significantly less than others.

All participants underwent a free screening program, including physical check-up, 13C breath test, upper gastrointestinal endoscopy, blood tests, and a doctor's interview. The study protocol was approved by the Ethical Committee of the people's Hospital of Yangzhong city. All participants received detailed written information about the study in advance and signed written informed consent before enrollment in the study.

### 2.2. Exclusion Criteria

The following individuals were excluded from our study: people taking medication for gastroesophageal reflux symptoms or malignant diseases; people with a history of *Helicobacter pylori* (*H. pylori*) eradication or upper gastrointestinal surgery. Individuals with gastric or esophageal cancer detected at the time of endoscopic screening were also excluded.

### 2.3. Questionnaires

This was a population-based study. All participants had been trained before they fulfilled the questionnaires. Collected information included sex, age, height, weight, individual education level, size of family, annual family income, marital status, self-reported socioeconomic group, and smoking and drinking habits. Health status, medical history, and medications taken in the past 2 months (particularly the use of proton pump inhibitors and antibiotics) were also recorded. Each questionnaire contained a total of 57 questions possibly related to *H. pylori *infection and transmission.

### 2.4. Detection of *H. pylori *Infection

Subjects were diagnosed with *H. pylori *infection if 13C-urea breath test (13C-UBT) was positive. The 13C-UBT was performed in the morning after at least 6 hours of fasting. The system is comprised of the following components: (a) a kit containing 50 mg of 13C-urea (a 99% 13C-enriched urea tablet); (b) a packet of granulated Citrica (a 4.5 gram packet containing 4 g of citric acid, 0.149 mg of aspartame, orange aroma, FD&C yellow #6); (c) an IDcircuit-sampling device; and (d) a BreathID device. All patients received 50 mg of 13C-urea with a 4.5 gram citric acid based powder (Citrica). The IDcircuit, a continuous nasal breath sampling device, transported the breath sample from the patient to the BreathID and did not require active cooperation. All performances followed the instructions. Our cut-off value is 4%, and we considered the patients negative when the value was less than 4% and positive when the value was larger than 4%.

### 2.5. Statistical Analysis

The data were recorded and analyzed by using EPIDATA 3.1 by double-input. Stata 12.0 was used for all statistical analyses. Multivariate analyses were restricted to those subjects with all relevant data available. A *χ* ^2^ test and Fisher's exact test of independence were used to compare the following variables of interest: sex, age, educational level, height, weight, pulse, blood pressure, size of family, annual family income, smoking, drinking, and the history of gastroenterology diseases. All the reported *P* values, are two-sided, and *P* value < 0.05 was regarded as statistically significant for all included studies. Logistic regression was used to select significant predictor variables and to estimate odds ratios (ORs) of these variables and, if possible, to predict outcomes.

## 3. Results

### 3.1. Prevalence of *H. pylori* Infection and Social Factors

Among 5500 subjects, 5417 completed both the questionnaire and the *H. pylori* detection test and were qualified for inclusion in data analysis. The mean age was 50.15 years old. There were 2342 men, whose average age was 50.27 years, and the rest were women, whose average age was 50.06 years. The overall prevalence of *H. pylori* was 63.41%. The prevalence of *H. pylori* among male and female was 61.74% and 64.47%, respectively, suggesting that there was a significant difference between sexes (*P* = 0.026) and women had a higher infection rate than men. As shown in Table 1, 30–39 years had the highest rate of *H. pylori* infection than other age groups.

The prevalence of *H. pylori* in the group with one member only (69.23%) was higher than that in the group with other members, without statistical difference. As for the association of prevalence and annual family income, in the six groups (with an average level of RMB 35890.95, approximately $US 5000), we found that individuals with an annual family income of RMB >80000 had the lowest risk of *H. pylori* infection (58.79%), whereas those with annual family income of RMB5000 or less had the highest risk of *H. pylori* infection (71.72%), and there was a significant difference between the groups (*P* = 0.017). Moreover, our study showed that the higher annual family income was, the lower the prevalence of *H. pylori*, which is significant in test for trend (*P* = 0.022). As for the education level, *H. pylori* infection is higher in illiterate (70.08%) and those who received university education had the lowest *H. pylori* infection rate (62.58%). But there were no significant associations seen for the subjects' level of education in general (*P* = 0.138).

Regarding the body mass index (BMI) value, healthy people (BMI = 18.5 to 24.9) had lowest *H. pylori* infection rate (62.28%) while those underweight (BMI < 18.5) people had highest infection rate (66.34%), but there was no significant difference between the groups.

There was no apparent association between the *H. pylori* infection and other social factors, such as marriage status (*P* = 0.369).

### 3.2. Relationships between *H. pylori *Infection and Dietary Factors, Smoking, and Drinking

The results suggest that *H. pylori* infection increased for subjects who ate vegetable more than once a day (65.71%), compared to those who ate it less than every other day (64.71%, ) and every other day to once a day (63.19% *P* = 0.309), and there was no difference in *χ* ^2^ for trend (*P* = 0.562), as shown in Table 2.

In contrast, the prevalence of *H. pylori* was highest in subjects who never ate fruits (67.70%), compared to the lowest infection rate in those who ate fruits less than every other day (63.35%, *P* = 0.45), and there was no significant difference in *χ* ^2^ for trend (*P* = 0.786).

Our study also showed that those who never ate milk, egg, or meat had 100% infection of *H. pylori* and people who ate less than every other day had the lowest infection rate (63.28%, *P* = 0.535). People who ate pickled food less than every other day had the highest infection (66.77%), while those who never ate pickled food had the lowest infection rate (62.39%, *P* = 0.032).

However, there was no apparent association between *H. pylori* infection and other dietary-related factors, such as beans consumption (*P* = 0.625), onion and garlic use (*P* = 0.926), eating fried foods (*P* = 0.065), and hot food (*P* = 0.526).

Table 3 shows that there was no association between the prevalence of *H. pylori* infection and the use of tobacco or alcohol (Table 3). But there was significant relationship observed between *H. pylori* infection and the amount of cigarettes which a person had been smoking per day. Our data showed that people smoking 1 to 10 cigarettes per day had significantly higher rate of *H. pylori* infection than people smoking 11 to 20 cigarettes (*P* = 0.041).

### 3.3. Relationships between *H. pylori *Infection and Upper Gastroduodenal Diseases

The prevalence of *H. pylori* infection in individuals with history of gastroenteral diseases (64.60%) was higher than those without a history of this disease (57.26%, *P* = 0.000) (Table 4).

### 3.4. Logistic Regression Model Analysis for *H. pylori* Infection

Thirty-six variables possibly related to *H. pylori* infection were assessed by using univariate logistic regression models analysis. The prevalence of *H. pylori* infection had a positive correlation with gender (*P* = 0.022), kipple food (*P* = 0.012), frequency of eating pickled food (*P* = 0.052), frequency of eating fried foods (*P* = 0.036), and digestive diseases (*P* = 0.000), and negative correlation with factors of age (*P* = 0.000), gastroenteral inflammation (*P* = 0.000) and peptic ulcer (*P* = 0.000) (Table 5(a)).

These eight factors then were introduced in the multivariate logistic regression analysis. Factors ultimately into the main effects model were independent factors for *H. pylori* infection. They were age (*P* = 0.000), history of gastroenteritis (*P* = 0.003), and history of peptic ulcer (*P* = 0.008) (Table 5(b)).

## 4. Discussion

To our knowledge, the present study was the first to demonstrate risk factors of *H. pylori* infection in Yangzhong country. However, five years ago, Shi et al. [9] also conducted a clinical trial investigating the prevalence of *H. pylori* infection in different areas, and compared to this previous study, our study found that the prevalence of *H. pylori* remained a continuously high rate in recent 5 years in Jiangsu province and that there were different risk factors related to *H. pylori* infection. The prevalence and onset of *H. pylori* in the general population are not clear since asymptomatic healthy individuals usually do not undergo endoscopic examination. Considering the large sample size and adjusting for various potential confounders, we believe that the results of this study accurately represent the risk factors of *H. pylori* infection in Yangzhong population.

In the present study, we determined an overall prevalence and investigated the risk factors of *H. pylori* in Yangzhong country. The prevalence of *H. pylori* infection varies all over the world, with less than 40% prevalence in developed countries and more than 80%–90% in developing countries [10]. The overall prevalence of *H. pylori* was 63.41% in Yangzhong city, similar to another previous study in Jiangsu Province by Shi et al. (62.07%) [9]. Since Yangzhong country is a high epidemic area of gastric cancer and lots of previous studies demonstrated that *H. pylori* infection can lead to gastric cancer [11, 12], our study was of great clinical importance. Recently, more attention has been given to the risk factors of *H. pylori* infection, such as gender, age, and socioeconomic status, but the result has still been controversial [13, 14].

Yangzhong is a relatively enclosed country with limited population shifts. So the prevalence of *H. pylori* infection is in its natural status. Thus, we randomly selected six regions in this country for the present study.

### 4.1. Prevalence of *H. pylori* Infection and Social Factors

Malcolm et al. [15] reported that the *H. pylori* infection was associated with age, sex, and socioeconomic conditions. In a previous study [9], there was no relationship between *H. pylori* and gender, age in adults, but annual family income was an important risk factor. Ariizumi et al. [16] found that the *H. pylori* infection rate was associated with age, but there was no statistical difference between *H. pylori* infection with gender and BMI. In Dore et al. study [17], no statistical difference was observed between different socioeconomic groups and age groups.

In our study, significant difference was observed in gender, age, and annual family income. We suggested that female had a higher infection rate than male. The explanation for this difference can only be speculative at this moment, being most probably related to the hormonal differences between the two genders, as recent studies identified an important role of oxytocin in the gastric evacuation rate (GER) [18]. Multivariate logistic regression model analysis also revealed that age was an important risk factor of *H. pylori* infection. The age group of 30–39 has shown a significant higher rate of *H. pylori* infection than other age groups. And the prevalence of *H. pylori* infection decreased with age. Previous studies showed that the infection rate was higher in childhood probably because people were usually infected with *H. pylori* when they were young [19]. A lower prevalence rate of *H. pylori* infection in the elderly has also been reported by others and two hypotheses have been proposed to explain these findings: *H. pylori* could have been present in a small number or low activation which might not have been detected. And *H. pylori* could have been present in the past, but was eliminated on account of the development of an unfavorable gastric environment with age. At the mean time, there is a progressive gastric migration in a proximal direction. As for the annual household income, we found that the prevalence increased when it was less than RMB5000 and the higher it was, the lower the prevalence of *H. pylori*, which was consistent with other reports [20, 21]. This might be related to the better living and sanitary conditions, with separate bedrooms for children.

The risk factors for *H. pylori* infection also include more family members, lower education level, and abnormal BMI. We found that there was no statistical different between each group. Among these factors, higher educational level was slight but not significant decrease with the *H. pylori* infection, which may be explained by their difference kinds of occupation. We also found that normal body mass index had lowest infection rate and those underweight had the highest rate probably due to their decreased nutritional status and immunity.

### 4.2. Relationships between *H. pylori *Infection and Dietary Factors

*H. pylori* infection could also be related to food and eating habits [22]. We investigated several dietary factors and found that eating kipper and fried food was positively associated with *H. pylori* infection. The consequence may be related to the way in which food is prepared, and dietary administration of salt may induce mucosal damage, such as diffuse erosion and degeneration, and destroy the mucosal barrier in the stomach. These changes in the gastric mucosa may be associated with an increased chance of persistent infection with *H. pylori*. Furthermore, salty food itself may be a source of *H. pylori*. The exact reason still needs to be further investigated.

### 4.3. Association between *H. pylori* and Drinking and Smoking

Two Japanese studies reported that smoking is negatively related to *H. pylori* infection [23, 24], but another study from Northern Ireland reported a positive relationship [25]. Other studies [26–29] found no relationship between smoking and *H. pylori* infection.

In our study, there was no association between the prevalence of *H. pylori* infection and the use of tobacco. And no significant relationship was observed between *H. pylori* infection and the period of time over which a person had been smoking, consistent with our previous study [9]. But in the present study, there was significant relationship observed between *H. pylori* infection and the amount of cigarettes smoked per day. Our data showed that people smoking 1 to 10 cigarettes per day had significantly higher rate of *H. pylori* infection than people smoking 11 to 20 cigarettes (*P* = 0.041), suggesting that the risk of *H. pylori* infection decreased with cigarette consumption per day.

The observed association of smoking with active *H. pylori* infection may result from various mechanisms with partly antagonistic effects on the risk of infection. Potentially relevant effects of smoking include an increase in acid and pepsin secretion and changes in gastric motility, prostaglandin synthesis, gastric mucosal blood flow, and mucus secretion [30].

As for drinking, our study showed that those who never drank beer had slightly higher rate of *H. pylori* infection than people who consumed mild and moderate beverage. Meanwhile, people never drinking wine had lower incidence of *H. pylori* infection. However, the difference has no significance.

Several previous studies have found the relation between *Helicobacter pylori* infection and alcohol consumption. Most of them did not find a significant association [31–35]. Interestingly, Brenner et al. [36] suggested a major protective effect of alcohol at moderate and high consumption but not at low consumption. Alcoholic beverages may directly and indirectly affect gastric mucosa, gastric acid secretion [37], and gastric emptying [38], leading to living condition changes of *H. pylori* in the stomach. In particular, moderate alcohol consumption might invigorate mucosal defense by its effects on prostaglandins [39]. Last, alcoholic beverages are known to have strong direct antibacterial activity [40–42].

But our study showed that there was no association between the prevalence of *H. pylori* infection and the use of alcohol, due to small sample size and heterogeneity between different groups. Besides consumption beverage, we also addressed other more details such as the type of alcoholic consumed and history of alcohol consumption. However there was no significant founding. Further studies involving larger numbers of subjects and multicenters should address more detailed additional factors and potential interactions between alcohol consumption and other factors that might affect active *H. pylori* infection.

### 4.4. Relationships between *H. pylori *Infection and Digestive Diseases

Shi et al. [9] found that there was no association between *H. pylori* infection and histories of upper gastrointestinal diseases. However, our present study showed that there was no association between *H. pylori* infection and hepatitis and esophagitis, but a positive correlation was found between peptic ulcer and gastroenteritis. The data were confirmed by multivariate logistic regression analysis.

*H. pylori* is motile, even in the highly viscous mucus layer. This may allow the organisms to evade both gastric motility and peristalsis, and also to some extent gastric acidity. Although it is motile, it also may adhere to the gastric mucosa through specific adhesion mechanisms. The secretion of large amounts of urease results in any urea in the environment being converted into ammonia-with the result that the intense acidity of the stomach may be ameliorated in the microenvironment surrounding the bacterium.

About 50% of *H. pylori* strains produce cytotoxins, of which some have been specifically linked to active gastritis and peptic ulceration. These cytotoxins can cause local inflammation, though other secretions by the organism, such as proteases and phospholipases, can attack and damage mucosal cell membranes. Weakening the gastric-mucosal barrier permits back-diffusion of hydrogen ions resulting in further tissue injury, as well as causing local immune responses to the organism.

## 5. Conclusion

In conclusion, the prevalence of *H. pylori* infection was 63.41% in the populations of Yangzhong country, Jiangsu province, which is also a region of high-risk gastric cancer in China. The prevalence of *H. pylori* infection was linked to sex, age, kipper food, frequency of eating kipper foods and fried food, peptic ulcer, and gastroenteritis. The relationship between *H. pylori* infection and other risk factors, such as upper gastrointestinal symptoms and some health habits, is still to be investigated.

## Figures and Tables

**Table 1:** Relationships between prevalence of *H. pylori* infection and general information.

Factors	No. of subjects in Hp positive group	No. of subjects in Hp negative group	Total no. of subjects	Prevalence	OR (95% CI)	*P* value
Sex						
Male	1446	896	2342	61.74%	1.00	1.000
Female	1989	1086	3075	64.68%	0.97 (0.87–1.08)	0.573
Age						
30–39	267	27	294	90.82%	1.00	1.000
40–49	1508	818	2326	64.83%	0.19 (0.13–0.28)	0.000
50–59	1374	921	2295	59.87%	0.16 (0.11–0.24)	0.000
60–69	285	215	500	57.00%	0.14 (0.09–0.22)	0.000
BMI						
Underweight	67	34	101	66.34%	1.00	1.000
Normal	2178	1319	3497	62.28%	0.87 (0.58–1.32)	0.516
Obese	1190	629	1819	65.42%	1.02 (0.67–1.55)	0.943
Marriage						
Unmarried	16	5	21	76.19%	1.97 (0.73–5.35)	0.183
Married	3326	1930	5256	63.28%	1.00	1.000
Divorced	27	18	45	60.00%	0.79 (0.44–1.44)	0.446
Loss of spouse	66	29	95	69.47%	1.32 (0.85–1.05)	0.217
Education level						
Illiterate	89	38	127	70.08%	1.38 (0.94–2.04)	0.100
Primary school	1824	1085	2909	62.70%	1.00	1.000
Middle school	1333	746	2079	64.12%	1.05 (0.94–1.18)	0.371
University or above	189	113	302	62.58%	0.94 (0.74–1.20)	0.617
Number of family members						
1	36	16	52	69.23%	1.00	1.000
2	237	151	388	61.08%	0.70 (0.37–1.30)	0.257
3	1000	602	1602	62.42%	0.74 (0.41–1.34)	0.320
4	491	280	771	63.68%	0.78 (0.42–1.43)	0.421
5	1466	820	2286	64.13%	0.79 (0.44–1.44)	0.449
6	152	83	235	64.68%	0.81 (0.43–1.55)	0.533
7 and above	53	30	83	63.86%	0.69 (0.25–1.92)	0.48
Annual income						
≤5000	104	41	145	71.72%	1.00	1.000
5001–20000	549	367	916	59.93%	0.81 (0.55–1.16)	0.249
20001–50000	2235	1248	3483	64.17%	0.86 (0.61–1.23)	0.418
50001–80000	450	258	708	63.56%	0.89 (0.61–1.30)	0.546
>80000	97	68	165	58.79%	0.78 (0.49–1.24)	0.299

**Table 2:** Relationships between prevalence of *H. pylori* infection and dietary-related factors.

Factors	No. of subjects in Hp positive group	No. of subjects in Hp negative group	Total no. of subjects	Prevalence of Hp	OR (95% CI)	*P* value
Frequency of eating vegetables						
<Every other day	22	12	34	64.71%	1.00	1.000
Every other day-once a day	3114	1814	4928	63.19%	0.94 (0.46–1.89)	0.853
>Once a day	299	156	455	65.71%	1.05 (0.51–2.19)	0.844
Frequency of eating fruits						
0	23	10	33	69.70%	1.00	1.000
<Every other day	3199	1851	5050	63.35%	0.64 (0.30–1.39)	0.261
Every other day-once a day	213	121	334	63.77%	0.72 (0.33–1.61)	0.427
Frequency of eating milk, egg, and meat						
0	2	0	2	100.00%	1.00	1.000
<Every other day	3211	1863	5074	63.28%	1.38 (1.01–1.88)	0.039
Every other day-once a day	113	58	171	66.08%	1.35 (0.88–2.09)	0.172
>Once a day	109	61	170	64.12%	1.00	
Frequency of eating beans						
0	8	3	11	72.73%	1.00	1.000
<Every other day	3229	1874	5103	63.28%	1.00 (0.29–3.43)	0.997
Every other day-once a day	198	105	303	65.35%	0.81 (0.23–2.84)	0.746
Frequency of eating onion and garlic						
0	11	6	17	64.71%	1.00	1.000
<Every other day	3271	1880	5151	63.50%	1.21 (0.46–3.19)	0.695
Every other day-once a day	146	92	238	61.34%	1.24 (0.45–3.37)	0.677
>Once a day	7	4	11	63.64%	0.84 (0.18–3.88)	0.823
Frequency of eating pickled foods						
0	2175	1311	3486	62.39%	1.00	1.000
<Every other day	448	223	671	66.77%	0.87 (0.73–1.03)	0.103
Every other day-once a day	810	447	1257	64.44%	0.97 (0.85–1.11)	0.683
>Once a day	2	1	3	66.67%	1.12 (0.10–12.44)	0.923
Frequency of eating fried foods						
0	3138	1826	4964	63.22%	1.00	1.000
<Every other day	294	155	449	65.48%	1.14 (0.93–1.40)	0.211
Every other day-once a day	3	1	4	75.00%	0.58 (0.08–4.14)	0.59
Frequency of eating hot foods						
0	3242	1856	5098	63.59%	1.00	1.000
<Every other day	191	125	316	60.44%	0.90 (0.71–1.14)	0.375
Every other day-once a day	2	1	3	66.67%	1.15 (0.10–12.66)	0.911

**Table 3:** Relationships between prevalence of *H. pylori* infection and smoking and alcohol drinking.

Factors	No. of subjects in Hp positive group	No. of subjects in Hp negative group	Total	Prevalence	OR (95% CI)	*P* value
*Smoking *						
No. of cigarettes smoked per day						
0	2691	1535	4226	63.68%	1.00	1.000
1–10	191	108	299	63.88%	0.86 (0.68–1.09)	0.218
11–20	509	298	807	63.07%	1.19 (1.01–1.40)	0.036
>20	44	41	85	51.76%	0.80 (0.52–1.23)	0.321
The period of smoking (year)						
0	2691	1535	4226	63.68%	1.00	1.000
0–10	186	105	291	63.92%	1.18 (0.92–1.52)	0.183
11–20	389	221	610	63.77%	1.14 (0.95–1.36)	0.152
>20	169	121	290	58.28%	0.88 (0.69–1.12)	0.286

*Drinking *	589	313	902	65.30%		
Beer	15	14	29	51.72%		
Beer consumption per day (mL/d)						
0	3420	1968	5388	63.47%	1.00	1.000
0–200	4	4	8	50.00%	0.96 (0.23–4.04)	0.960
201–500	9	10	19	47.37%	2.17 (0.72–6.54)	0.170
>500	2	0	2	100.00%	0.58 (0.04–9.25)	0.699
The period of drinking beer (year**)**						
0	3420	1968	5388	63.47%	1.00	1.000
0–10	10	9	19	52.63%	2.17 (0.72–6.54)	0.169
11–20	5	2	7	71.43%	0.77 (1.72–3.45)	0.734
>20	0	3	3	0.00%	1.74 (0.18–16.69)	0.633
Wine	577	301	878	65.72%		
Wine consumption per day (g/d)						
0	2858	1681	4539	62.97%	1.00	1.000
<250	480	248	728	65.93%	1.07 (0.90–1.25)	0.445
251–500	95	53	148	64.19%	0.96 (0.68–1.34)	0.791
>500	2	0	2	100.00%		
The period of drinking wine (year)						
0	2858	1681	4539	62.97%	1.00	1.000
0–10	186	110	296	62.84%	1.16 (0.90–1.48)	0.250
11–20	275	136	411	66.91%	1.04 (0.85–1.29)	0.686
>20	116	55	171	67.84%	0.90 (0.66–1.23)	0.521

**Table 4:** Relationships between prevalence of *H. pylori* infection and history of digestive diseases.

Factors	No. of subjects in Hp positive group	No. of subjects in Hp negative group	Total no. of subjects	Prevalence of Hp	OR (95% CI)	*P* value
History of digestive diseases	501	374	875	57.26%	0.71 (0.61–0.82)	0.000
Gastroenteritis	324	262	586	55.29%	0.67 (0.57–0.80)	0.000
Peptic ulcer	91	87	178	51.12%	0.57 (0.42–0.76)	0.000
Esophagitis	53	32	85	62.35%	0.95 (0.61–1.49)	0.838
Hepatitis	73	46	119	61.34%	0.91 (0.63–1.33)	0.636

**Table:** (a)

Risk factors	*B*	Sx	Wald	*P* value	OR	95% CI
Gender	0.13	0.057	5.207	0.022	1.14	1.01–1.27
Age	−0.037	0.004	78.336	0.000	0.96	0.95–0.97
Marriage	0.073	0.1	0.527	0.468	1.08	0.88–1.31
Education level	−0.011	0.044	0.064	0.800	1.00	0.91–1.08
Family number	0.013	0.02	0.436	0.570	1.01	0.97–1.05
Annual income	0.000	0.000	0.212	0.748	1.00	1.00–1.00
Smoking	−0.076	0.08	0.902	0.722	0.98	0.85–1.11
Number of cigarettes per day	0.003	0.003	0.934	0.347	1.00	0.99–1.01
The period of smoking	−0.059	0.036	2.643	0.356	0.97	0.91–1.03
Alcohol	0.108	0.09	1.458	0.404	1.66	0.92–1.24
Beer consumption per day	0.000	0.001	0.039	0.455	1.00	1.00–1.00
The period of drinking beer	−0.033	0.037	0.768	0.220	0.97	0.93–1.02
Wine consumption per day	0.001	0.001	0.862	0.208	1.00	1.00–1.00
The period of drinking beer	0.003	0.007	0.177	0.333	1.00	0.99–1.01
Tea	−0.043	0.137	0.099	0.831	0.97	0.74–1.27
Frequency of eating vegetables	0.002	0.003	0.488	0.485	1.00	0.99–1.01
Fruit	−0.166	0.184	0.812	0.336	0.84	0.58–1.20
Frequency of eating fruits	−0.001	0.005	0.011	0.918	1.00	0.99–1.01
Meat, milk, and eggs	−2.527	3.536	0.511			
Frequency of eating milk, egg, and meat	0.002	0.003	0.31	0.577	1.00	0.99–1.01
Bean	−0.395	0.715	0.305	0.524	0.65	0.17–2.45
Frequency of eating beans	0.002	0.006	0.151	0.698	1.00	0.99–1.01
Onion and garlic	−0.059	0.42	0.02	0.738	0.88	0.41–1.89
Frequency of eating onion and garlic	0.011	1.007	2.516	0.112	1.01	1.00–1.02
Kipper food	0.164	0.065	6.349	0.012	1.16	1.03–1.30
Frequency of eating pickled foods	0.006	0.003	3.774	0.052	1.01	1.00–1.02
Fried food	0.183	0.132	1.931	0.245	1.13	0.92–1.38
Frequency of eating fried foods	0.073	0.035	4.309	0.036	1.08	1.00–1.15
Scalding food	−0.347	0.147	5.568	0.321	0.89	0.70–1.12
Frequency of eating hot foods	0.009	0.033	0.072	0.788	1.01	0.95–1.08
Digestive diseases	0.171	0.207	0.683	0.000	0.71	0.61–0.82
Gastroenteral inflammation	−0.523	0.206	6.457	0.000	0.67	0.57–0.80
Peptic ulcer	−0.64	0.217	8.706	0.000	0.57	0.42–0.76
Esophageal inflammation	−0.047	0.226	0.044	0.838	0.95	0.61–1.49
Hepatitis	−0.239	0.26	0.85	0.636	0.91	0.63–1.33
BMI	−0.001	0.001	0.685	0.426	1.00	1.00–1.00

**Table:** (b)

Risk factors	*B*	S.E.	Wald	*P* value	OR	95% CI
Gender	0,112	0.058	3.756	0.053	1.12	1.00–1.25
Age	−0.04	0.004	88.709	0.000	0.96	0.95–0.97
Kipper food	0.142	0.115	1.524	0.217	1.15	0.92–1.44
Frequency of eating pickled foods	−0.003	0.006	0.254	0.614	1.00	0.99–1.01
Frequency of eating fried foods	0.045	0.035	1.697	0.193	1.05	0.98–1.12
Digestive disease	0.131	0.155	0.705	0.401	1.14	0.84–1.54
Gastroenteritis	−0.493	0.166	8.795	0.003	0.61	0.44–0.85
Peptic ulcer	−0.51	0.193	7.001	0.008	0.6	0.41–0.88
